# Immunological Mechanisms Underlying Allergy Predisposition After SARS-CoV-2 Infection in Children

**DOI:** 10.3390/cells14191511

**Published:** 2025-09-28

**Authors:** Filippos Filippatos, Dimitra-Ifigeneia Matara, Athanasios Michos, Konstantinos Kakleas

**Affiliations:** First Department of Pediatrics, Medical School, National and Kapodistrian University of Athens, “Aghia Sophia” Children’s Hospital, 11527 Athens, Greece; kontifi@gmail.com (D.-I.M.); amichos@med.uoa.gr (A.M.); koskakl2@yahoo.gr (K.K.)

**Keywords:** SARS-CoV-2, COVID-19, allergy, immunity, cytokines

## Abstract

As the pediatric COVID-19 landscape evolves, it is essential to evaluate whether SARS-CoV-2 infection predisposes children to allergic disorders. This narrative review synthesizes current epidemiological and immunological evidence linking pediatric COVID-19 with new-onset atopy. Epidemiological data remain heterogeneous: large Korean and multinational cohorts report increased risks of asthma and allergic rhinitis following COVID-19, whereas U.S. cohorts show neutral or protective associations, highlighting geographic and methodological variability. Mechanistic insights provide biological plausibility: epithelial injury and the release of alarmin cytokines (IL-33, IL-25, TSLP) promote Th2 polarization and ILC2 expansion, while epigenetic “scars” (e.g., LMAN2 methylation changes) and hematopoietic stem cell reprogramming may sustain long-term Th2 bias. Cytokine memory involving IL-7 and IL-15 contributes to altered T- and B-cell homeostasis, whereas disrupted regulatory T-cell function may reduce tolerance thresholds. Paradoxical trade-offs exist, such as ACE2 downregulation in allergic airways, which may lower viral entry but simultaneously amplify type-2 inflammation. Together, these processes suggest that SARS-CoV-2 infection could foster a pro-allergic milieu in susceptible children. Although current evidence is inconclusive, integrating epidemiological surveillance with mechanistic studies is crucial for predicting and alleviating post-COVID allergic outcomes. Longitudinal pediatric cohorts and interventions targeting epithelial alarmins or microbiome restoration may hold promise for prevention.

## 1. Introduction

SARS-CoV-2 infections in children are generally mild or asymptomatic, suggesting robust antiviral immunity [1,2]. Children constitute a disproportionately minor number of severe COVID-19 cases and fatalities, with most pediatric infections resulting in extremely mild symptoms. However, rare outcomes—such as multisystem inflammatory syndrome in children (MIS-C)—highlight that SARS-CoV-2 can provoke post-infectious immunological dysregulation in the pediatric demographic [3]. The data suggest that while children often handle acute illnesses effectively, the recovery immunological milieu may be altered in ways that could affect long-term immune homeostasis [4].

Respiratory viral infections are well-established contributors to allergy development [5]. Infections during early infancy, such as RSV or rhinovirus bronchiolitis, often precede asthma, and animal studies suggest that respiratory viruses can enhance sensitization to aeroallergens. Epidemiological observations consistently suggest that viral-induced epithelial barrier disruption and skewed cytokine responses may lower the threshold for allergic sensitization in genetically predisposed children [6,7]. Consequently, it is critical to investigate whether SARS-CoV-2, a virus with unique epithelial and immunomodulatory effects, may act as a novel trigger for pediatric atopy [8].

Understanding this association holds major clinical significance. Childhood asthma and allergic diseases impose substantial global health and economic burdens, and identifying modifiable post-viral risk factors could inform preventive strategies. Recent studies indicate that children recovering from COVID-19 may face increased risks of new-onset asthma and allergic rhinitis, although findings remain inconsistent across geographical regions. These epidemiological signals align with mechanistic evidence that SARS-CoV-2 alters the Th1/Th2/Treg axis, disrupts tolerance checkpoints, and induces long-lasting epigenetic scars in immune progenitors.

Viral-induced epithelium “alarmin” cytokines and innate type 2 effectors can mechanistically skew immunity towards allergies [9]. The diseased airway epithelium secretes IL-33, IL-25, and thymic stromal lymphopoietin (TSLP), which are powerful activators of ILC2 and Th2 mast cell pathways [10]. TSLP is up regulated by NF-κB in response to infection or injury, and IL-33 is released following epithelial damage [11]. These alarmins regulate crucial type 2 cells, including ILC2, Th2 cells, mast cells, basophils, and dendritic cells [12]. Rhinovirus infection in murine airways stimulates TSLP, IL-33, and OX40L, suppresses the production of Foxp3^+^ T regulatory cells, and sustains Th2-biased allergic inflammation [13]. The pathways produced by viruses involving TSLP/IL-33–OX40L can impair tolerance and exacerbate allergic inflammation [14].

This review examines the biological plausibility and clinical relevance of allergy predisposition following pediatric SARS-CoV-2 infection. By integrating epidemiological data with immunological insights, including epithelial alarmins, Th2 polarization, cytokine memory, and microbiome changes, we aim to clarify whether COVID-19 represents an emerging risk factor for atopy in children. Addressing this question is essential not only for prognostication but also for developing targeted interventions to mitigate long-term allergic outcomes.

## 2. Incidence of Post–COVID New-Onset Allergies or Asthma

Emerging epidemiological evidence not only supports the paramount increase in COVID-19 pediatric cases [15] but also provides critical context for interpreting mechanistic data linking SARS-CoV-2 infection with new-onset asthma or allergic disorders, particularly in pediatric populations. In a nationwide Korean National Health Insurance Service (NHIS) cohort encompassing approximately 150,000 pediatric COVID-19 survivors matched with nearly 750,000 uninfected children, 1.6% developed incident asthma within one year versus 0.7% of controls (31.3 vs. 14.6 cases per 1000 person-years; adjusted hazard ratio [aHR] 2.14, 95% CI 1.88–2.45), clearly suggesting a significant viral-induced risk [16]. Further bolstering these findings, Oh et al. conducted a comprehensive multinational analysis (>22 million electronic health records and biobank data) from South Korea, Japan, and the UK, reporting an overall 20% increased risk (aHR 1.20, 95% CI 1.13–1.27) of any allergic disease post-COVID-19, with specific elevations observed for asthma (aHR 2.25, 95% CI 1.80–2.83) and allergic rhinitis (aHR 1.23, 95% CI 1.15–1.32). Notably, risks persisted for at least six months post-infection and scaled with acute COVID-19 severity, while ≥2 vaccine doses conferred protective effects, lowering allergic-disease incidence below baseline (aHR 0.81, 95% CI 0.68–0.96) [17].

In contrast, large pediatric cohorts from the United States present a more heterogeneous picture. Kompaniyets et al. analyzed 781,419 pediatric COVID-19 cases via U.S. commercial claims data (March 2020–January 2022), identifying increased post-COVID risks predominantly for myocarditis (aHR 1.99), thromboembolic events (aHR 1.87), and diabetes (aHR 1.31), but did not detect increased asthma diagnoses, possibly reflecting differences in healthcare access or regional pathogen exposure patterns [18]. A dedicated prospective cohort study from the Children’s Hospital of Philadelphia similarly found no association between COVID-19 and asthma onset in a carefully controlled analysis of 15,000 infected and matched controls (aHR 0.96, 95% CI 0.73–1.25, *p* = 0.79) [19]. Additionally, ecologic data derived from U.S. claims databases indicated that incident pediatric asthma diagnoses nationally fell substantially during the COVID-19 pandemic period (adjusted incidence rate ratio [aIRR] 0.47, 95% CI 0.43–0.51), presumably due to reduced circulation of typical viral triggers such as rhinovirus and respiratory syncytial virus, and decreased healthcare utilization rather than protective effects of SARS-CoV-2 itself [20].

Complementary international data further contextualize these observations. Recent UK Biobank analyses revealed modestly elevated risks for allergic respiratory conditions in adults post-COVID-19, echoing pediatric trends (aHR 1.11–1.26 across conditions), suggesting age-independent immune-mediated processes [21]. Similarly, German pediatric outpatient studies showed subtle yet statistically significant upticks in atopic conditions (asthma, allergic rhinitis) after SARS-CoV-2 infection, particularly pronounced after severe disease courses [22]. Conversely, Scandinavian nationwide pediatric registries have not consistently identified excess allergic outcomes post-COVID-19, highlighting geographical and methodological heterogeneity [23].

Collectively, these data underscore that while absolute risks of new-onset allergic disease following pediatric SARS-CoV-2 infection remain modest, there is accumulating evidence from multiple international cohorts of a vaccine-modifiable increase in asthma and related atopic disorders. These epidemiological signals strongly align with mechanistic hypotheses implicating SARS-CoV-2-induced epithelial barrier disruption, dysregulated Th2 and innate lymphoid cell responses, and enhanced IgE-mediated sensitization pathways [24].

## 3. Immunological Mechanisms

**Figure 1:**
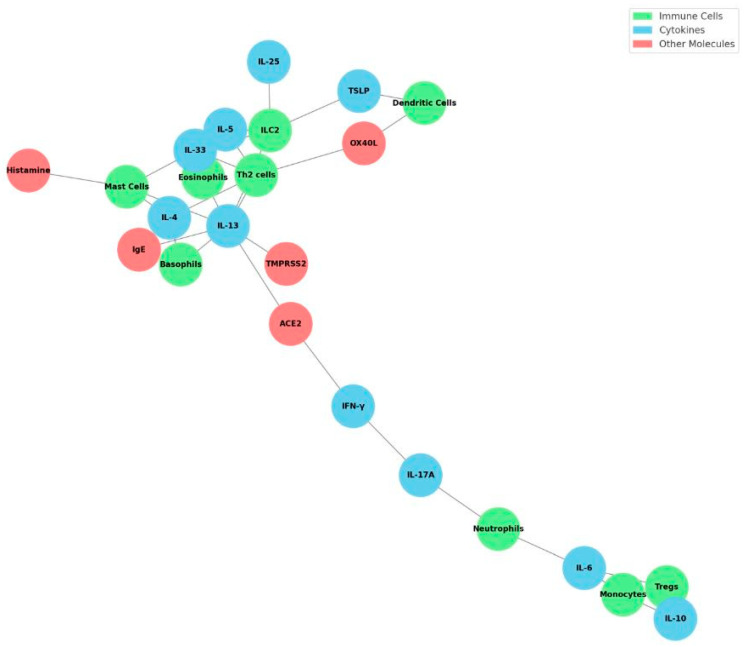
Network of Cells, Cytokines, and Molecules in COVID-19 Allergy Immunopathology. This network diagram visually summarizes the complex interactions between key immune cells, cytokines, and other molecules involved in the immunopathogenesis of allergy predisposition following SARS-CoV-2 infection. Immune cells (green nodes) such as type 2 innate lymphoid cells (ILC2), Th2 cells, eosinophils, mast cells, basophils, regulatory T cells (Tregs), monocytes, dendritic cells, and neutrophils are interconnected through cytokines (blue nodes), including IL-33, IL-25, thymic stromal lymphopoietin (TSLP), IL-4, IL-5, IL-13, IL-10, IFN-γ, IL-17A, and IL-6. Other critical molecules (red nodes) such as IgE, ACE2, TMPRSS2, OX40L, and histamine further bridge these interactions, reflecting molecular mechanisms that drive type 2 immune skewing and allergic sensitization after SARS-CoV-2 infection. This figure highlights potential therapeutic targets and underscores the interconnected nature of antiviral responses, epithelial barrier dysfunction, innate immune activation, and allergic pathways.

The interplay between antiviral and pro-allergic immune networks is summarized in Figure 1, which provides an overview of the immunological crosstalk between SARS-CoV-2 infection and allergy predisposition.

Allergic diseases in children are characterized by type 2 (Th2) immunity, defined by IL-4, IL-5, IL-13 production, IgE class switching, and eosinophil/mast cell activation [25,26]. SARS-CoV-2 alters these networks by disrupting the Th1/Th2/Treg balance [27] and modulating epithelial receptors ACE2 and TMPRSS2 [28,29,30,31]. In atopic children, ACE2 expression is downregulated experimentally in vivo [29,30], while IL-13 exposure reduces ACE2 and increases TMPRSS2 transcripts in bronchial epithelium. These reciprocal effects suggest that viral entry and replication may differ in allergic airways, creating context-dependent trade-offs.

Eosinophils, elevated in allergic children [32], display antiviral properties through granule proteins, nitric oxide, and type I cytokines while recruiting CD8^+^ T cells [33,34]. Eosinopenia is a biomarker of severe adult COVID-19, whereas allergic children infected with SARS-CoV-2 exhibit higher eosinophil counts [35], potentially explaining their relatively mild disease [36].

Resistance against SARS-CoV-2 requires early type I/III interferon release and Th1 cytotoxicity [37]. Efficient antiviral defense in children also relies on type I and III interferons (IFN-α/β and IFN-λ), which are produced more robustly in pediatric airways than in adults. Several studies have demonstrated that children exhibit a pre-activated antiviral state, with higher basal expression of pattern recognition receptors such as MDA5 and RIG-I, leading to faster and stronger IFN-I/III induction upon SARS-CoV-2 infection [37]. This heightened interferon activity is associated with rapid viral clearance and likely contributes to the typically mild course of pediatric COVID-19. By contrast, adults often display delayed or impaired IFN-I/III responses, sometimes compounded by neutralizing anti-IFN autoantibodies, predisposing them to severe disease. The enhanced interferon competence of children therefore represents a critical protective mechanism against SARS-CoV-2, counterbalancing Th2-associated vulnerabilities and shaping long-term immune memory [38]. Th2 cytokines can counterbalance these responses: IL-4/IL-13 inhibit interferon signaling, while a Th2-skewed environment may protect from cytokine storm-driven lung injury [39,40]. This illustrates a duality where allergic inflammation may both attenuate viral clearance and promote sensitization [41,42].

Children with allergies display hyperresponsive mast cells; nonetheless, the function of histamine in the pathobiology of COVID-19 remains speculative [43,44]. In vitro studies indicate that the histamine H1 receptor (HRH1) may function as a viral co-receptor, and H1 antihistamines inhibit the entry of SARS-CoV-2. Limited clinical studies in adults indicate symptomatic enhancement with cetirizine or famotidine; however, robust pediatric trials are deficient [45]. Regular antihistamine administration has not exacerbated outcomes in allergic children and may provide slight alleviation of upper airway symptoms, although the evidence remains preliminary [46,47,48]. Overall, pediatric allergy inflammation appears to reduce SARS-CoV-2 receptor availability and support eosinophil-mediated viral control, but sustained Th2 bias may inhibit interferon pathways and increase long-term risk of allergy [49,50,51,52,53,54].

Severe infections induce lasting epigenetic alterations in monocytes and progenitors—termed trained immunity—that reprogram cytokine responses long after recovery [53]. This imprinting can sustain hyper-responsive ILC2 activity and a pro-Th2 program, increasing atopy risk [54].

Type 2 cytokines further influence airway vulnerability: IL-4 and IL-13 downregulate ACE2 while upregulating TMPRSS2 in pediatric epithelial cells [55,56,57]. IFN-γ exerts the opposite effect, enhancing ACE2 expression [58], illustrating context-dependent regulation of viral entry [59]. In addition to cytokine-mediated suppression by IL-4 and IL-13, recent studies have demonstrated that ACE2 is an interferon-stimulated gene (ISG). IFN-γ and type I interferons upregulate ACE2 expression in airway epithelial cells [57], supporting its classification as an ISG. This creates an apparent paradox: while type 2 cytokines (IL-4/IL-13) reduce ACE2 and increase TMPRSS2 [54,55,56], interferon signaling enhances ACE2 availability [57]. The context of cytokine dominance therefore determines receptor biology—interferon-rich environments favor viral entry via ACE2, whereas Th2-skewed airways limit entry but may enhance allergic sensitization through epithelial alarmins. Recognizing ACE2 as an ISG thus helps reconcile these seemingly contradictory pathways in SARS-CoV-2 infection and atopy predisposition.

Atopic airways harbor abundant ILC2s that release IL-5/IL-13 upon IL-25, IL-33, or TSLP stimulation [60], while severe pediatric COVID-19 is marked by ILC depletion, with higher baseline ILC levels linked to reduced hospitalization risk [61]. Peripheral eosinophilia correlates with milder disease, whereas eosinopenia signals severity [62]. Mast cells, central in allergy, also contribute antiviral interferons but can be activated by SARS-CoV-2 spike protein, bridging allergic and antiviral pathways [63]. Finally, children exhibit higher frequencies of Treg and Breg cells compared to adults, which may mitigate hyperinflammation and support neutralizing antibody generation [64]. Collectively, a Th2/ILC2/eosinophil-dominant milieu may protect against severe interferon-driven pathology while subtly reshaping long-term immune memory [65].

## 4. Immunopathogenesis: Cytokines, Receptors, and Immune Cells

The immunopathogenesis of post-COVID atopy involves cytokine crosstalk, innate lymphoid cells, and immune tolerance checkpoints. While Th2 cytokines dominate allergy [66,67], respiratory viruses also induce Th1/Th17 cytokines (IFN-γ, IL-17), with epithelial alarmins (IL-33, IL-25, TSLP) paradoxically amplifying ILC2 responses [68]. SARS-CoV-2 reduces Treg number and function in both acute and convalescent samples [68,69], undermining tolerance and promoting sensitization.

Severe infection imposes lasting epigenetic changes in monocytes and progenitors (“trained immunity”), skewing cytokine production [70] and reinforcing pro-Th2 transcriptional programs [71]. Pediatric surveillance should therefore evaluate Th1/Th2/Th17/Treg balance, ILC activation, tolerance checkpoints, and epigenomic signatures [72].

Type-2 cytokines significantly modify SARS-CoV-2 receptor biology. IL-4/IL-13 reduce ACE2 and increase TMPRSS2 [73,74], confirmed by pediatric biopsy analyses [75]. Interferons exert opposite effects: IFN-γ upregulates ACE2 [76,77], while IL-4/IL-13 inhibit it [78]. These contrasting influences underscore context-dependent viral entry dynamics in atopic airways [76,79].

ILC2 expansion in allergic children (driven by IL-25, IL-33, TSLP) produces IL-5/IL-13 [80,81,82], whereas severe pediatric COVID-19 shows ILC depletion, with baseline ILC abundance linked to reduced hospitalization [80,81,82]. Peripheral eosinophilia correlates negatively with COVID-19 severity, while mast cells contribute both antiviral interferons and pro-inflammatory mediators [83].

Regulatory T cells normally maintain tolerance [84], but MIS-C demonstrates Notch-1/CD22-mediated Treg destabilization [85]. SARS-CoV-2 also induces epigenetic hypomethylation at IFN-stimulated genes and at the LMAN2 locus, connecting viral infection with Th2 skewing [86,87].

IL-7 and IL-15 are pivotal survival factors for memory T-cell pools, ensuring long-term maintenance of CD4^+^ and CD8^+^ subsets after viral infection. IL-7 primarily supports the survival of naïve and central memory T cells through the upregulation of Bcl-2, while IL-15 drives homeostatic proliferation and sustains effector memory CD8^+^ T cells [52,53,85].

During acute SARS-CoV-2 infection, dysregulated production of IL-7 and IL-15 has been reported, with transient elevations followed by variable normalization in recovery cohorts. When balanced, IL-7 and IL-15 ensure a resilient antiviral memory pool that supports rapid IFN-γ release upon re-exposure [52,53,85]. However, if their regulation is perturbed, several consequences may arise: 1. Overactive IL-15 signaling can drive bystander proliferation of heterologous CD8^+^ and CD4^+^ memory T cells, increasing the risk of nonspecific activation and tissue inflammation. 2. Insufficient IL-7 availability during recovery may impair Treg renewal, lowering tolerance thresholds and allowing allergen-reactive clones to persist. 3. Excess IL-15 in a Th2-biased cytokine milieu (rich in IL-4 and IL-13) may paradoxically expand memory cells with type-2 functional polarization, thus promoting allergic sensitization [52,53,85].

In children, where the immune system is still undergoing maturation, even subtle alterations in cytokine memory can disproportionately influence long-term immune homeostasis. Dysregulated IL-7/IL-15 signaling after pediatric COVID-19 may therefore contribute to a skewed memory repertoire that favors Th2/allergic responses over balanced antiviral recall. Any post-COVID surveillance of pediatric atopy must thus assess not only Th1/Th2/Th17/Treg balance, ILC activation, checkpoint integrity, and epigenomic state, but also the cytokine memory network mediated by IL-7 and IL-15. These axes may represent key mechanistic links between viral recovery and allergy predisposition.

The airway of an allergic child is predisposed to Th2/ILC2/eosinophil biology, providing protection against severe interferon-mediated pathology while subtly altering antiviral memory; the development of new-onset atopy will depend on the interaction of alarmins, interferons, and epigenetic memory in the months after the clearance of SARS-CoV-2 [88,89].

Although certain mechanisms may appear contradictory—such as the downregulation of ACE2 expression in allergic airways, which theoretically reduces viral entry, versus the simultaneous amplification of epithelial alarmin release and Th2 cytokine signaling—these findings are best understood as context-dependent trade-offs rather than mutually exclusive outcomes. Reduced ACE2 and TMPRSS2 expression in atopic children or in those receiving inhaled corticosteroids may transiently limit viral docking; however, epithelial injury from SARS-CoV-2 still induces IL-33, IL-25, and TSLP, which drive ILC2 and Th2 polarization. In this setting, the loss of interferon-mediated antiviral tone further permits exaggerated type-2 responses, promoting allergic sensitization despite fewer entry receptors. Thus, SARS-CoV-2 infection may simultaneously decrease infection susceptibility in atopic patients while priming a microenvironment that favors long-term Th2 skewing and atopic predisposition.

## 5. Immunological and Biological Mechanisms

**Figure 2:**
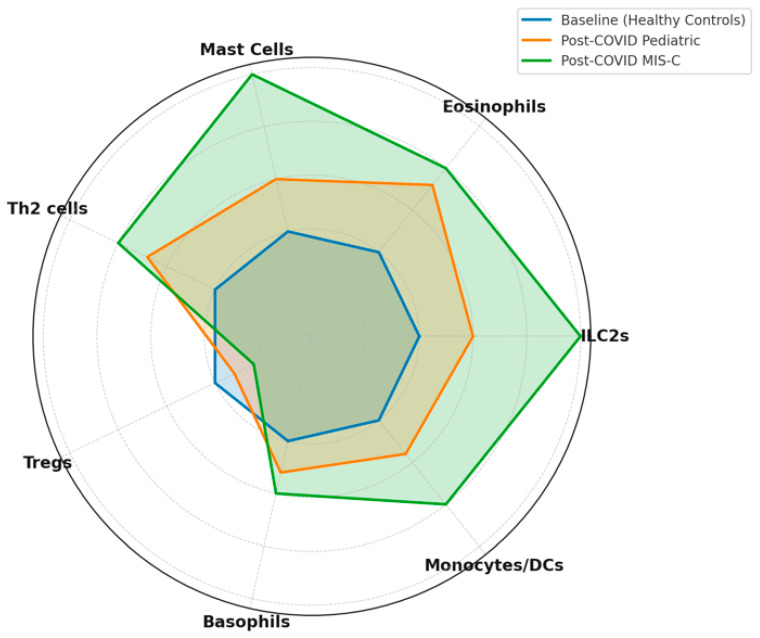
Cellular Changes Post-SARS-CoV-2 Linked to Allergy Risk. This radar chart illustrates the relative changes in key immune-cell populations associated with allergic predisposition following SARS-CoV-2 infection in children. It compares baseline immune cell levels (healthy controls), general pediatric patients post-COVID-19, and pediatric patients with multisystem inflammatory syndrome (MIS-C). Notably, post-COVID pediatric populations exhibit increased populations of innate lymphoid cells type 2 (ILC2s), eosinophils, mast cells, Th2 cells, basophils, and monocytes/dendritic cells (DCs), alongside a decrease in regulatory T cells (Tregs). These cellular shifts are markedly more pronounced in MIS-C patients, underscoring the potential impact of severe COVID-19-associated inflammation on long-term immune alterations favoring allergic responses. This visualization highlights critical immune-cell dynamics that could contribute to an increased risk of developing allergic diseases after SARS-CoV-2 infection. Charts were generated in Python 3.12 (matplotlib) and exported at 600 dpi (PNG). Schematic/qualitative visualizations (not a meta-analysis), using relative scales (0–5) informed by the directionality and magnitude reported across cited studies; they are intended to communicate comparative patterns across disease states (mild pediatric COVID-19, MIS-C, recovery), not pooled effect sizes.

Multiple biological pathways have been proposed to link SARS-CoV-2 infection with subsequent allergy risk. Acute SARS-CoV-2 infection—especially MIS-C—is characterized by elevated IL-6, IL-17A, and IL-18 [89,90], followed by persistently increased IL-10 that promotes Th2 polarization [91]. IL-13, central to asthma pathogenesis, correlates with pediatric COVID-19 severity, and its blockade improves survival in models [92], suggesting that SARS-CoV-2 can prime Th2 circuits [93] (Figure 2).

Epigenome-wide studies reveal CpG changes at the LMAN2 locus, favoring Th2 immunity [94]. Microbiome alterations further disrupt tolerance: children show Pseudomonas-dominated dysbiosis with loss of Bifidobacterium and Akkermansia [95,96], while MIS-C is associated with profound depletion of Faecalibacterium prausnitzii [97]. Pandemic-era cohorts also display reduced microbial diversity, a known predictor of later atopy [98,99,100].

Genetics modulate outcomes, with interferon-signaling alleles shaping COVID-19 severity [100,101,102]. Children with inborn errors of immunity manifest both severe COVID-19 and allergic phenotypes [103]. Beyond the acute phase, T-cell exhaustion and innate hyperactivation persist [104,105], potentially impairing tolerance and exaggerating Th2 responses upon allergen exposure [106]. Analogous viral–allergy links are known for measles and EBV [107], supporting the plausibility of SARS-CoV-2 as a driver of post-infectious atopy.

## 6. Cytokine Profiles and Persistent Inflammation

**Figure 3:**
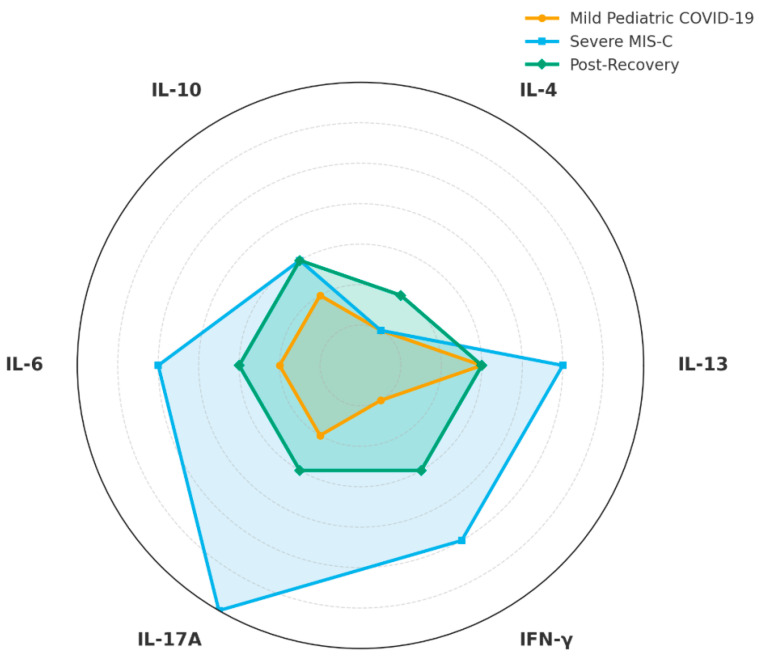
Th1/Th2/Th17/Treg Cytokine Dynamics during and after SARS-CoV-2 Infection. This radar chart summarizes relative cytokine levels representative of Th1 (IFN-γ), Th2 (IL-4, IL-13), Th17 (IL-17A), and regulatory T cell-associated cytokines (IL-10), alongside IL-6, during three different phases of pediatric SARS-CoV-2 infection. Mild pediatric COVID-19 infection shows moderate Th2 cytokine elevation and relatively balanced immune responses. Severe multisystem inflammatory syndrome in children (MIS-C) is distinguished by prominent elevations in IL-17A, IL-13, IFN-γ, IL-6, and IL-10, reflecting a robust hyperinflammatory state. During the post-recovery phase, cytokine profiles suggest persistent mild-to-moderate inflammation with sustained Th2 skewing (IL-13 elevation) and mild IL-17A expression. This figure highlights how cytokine dynamics differ substantially across clinical severity and recovery states, offering insights into potential immunological mechanisms underlying post-infectious allergy predisposition in pediatric patients following COVID-19. Charts were generated in Python 3.12 (matplotlib) and exported at 600 dpi (PNG). Schematic/qualitative visualizations (not a meta-analysis), using relative scales (0–5) informed by the directionality and magnitude reported across cited studies; they are intended to communicate comparative patterns across disease states (mild pediatric COVID-19, MIS-C, recovery), not pooled effect sizes.

Chronic cytokine dysregulation is a hallmark of post-COVID immunological sequelae and differs among mild pediatric COVID-19, severe MIS-C and post-recovery patients, with several cytokines strongly linked to allergy pathways [108,109] (Figure 3).

SARS-CoV-2 infection results in increased levels of IL-6, IL-1β, and TNF-α during the acute phase, and some individuals do not fully normalize after recovery [108]. Children suffering from post-COVID multisystem inflammation or prolonged COVID often exhibit sustained increases in IL-6 and other innate mediators [110,111,112,113]. Chronic IL-6 can skew B cells towards plasmablast differentiation and enhance IgE production [114,115,116].

IL-10, an anti-inflammatory cytokine that is paradoxically increased in MIS-C, can also promote IgG4 and IgE switching in Th2-polarized conditions [117]. SARS-CoV-2 has been shown to elicit virus-specific IgE in select individuals; one study suggested that even moderate infections stimulated IgE production against the viral spike protein, indicating the participation of allergic-type immune responses [118,119]. Notably, IgE responses were detected following illness or immunization, especially after booster doses [118,119]. The observations indicate that SARS-CoV-2 antigens may provoke class-switching to the IgE isotype, possibly via Th2-biased pathways [118,119].

If these virus-induced IgE responses signify immunological flexibility, similar skewing may occur upon exposure to environmental allergens subsequent to infection [109]. Other cytokines relevant to allergy are thymic stromal lymphopoietin (TSLP), IL-25, and IL-33, which are released by damaged epithelial cells and facilitate type 2 inflammation [113,114]. Recent studies suggest that COVID-19 can compromise epithelial barriers in the lungs and stomach, resulting in an increase in alarmins such as IL-33 [115]. Increased levels of IL-33 and its receptor ST2 in MIS-C suggest that tissue damage and Th2-promoting signals may persist following infection [110,112,115]. The cytokine milieu following SARS-CoV-2 infection, characterized by enduring antiviral (IL-6, IFN-γ) and reparative/alarmin (IL-10, IL-33, IL-13) signals, may cultivate a pro-allergic environment [108,109,115]. Chronic low-grade inflammation can enhance dendritic cell costimulatory activity and exacerbate Th2 priming upon re-exposure to allergens in children, eventually increasing allergic sensitization and the persistence of disease [108,109].

## 7. Regulatory T Cells and the Disruption of Tolerance

Regulatory T cells (Tregs) are essential for sustaining immunological tolerance and averting allergies [120]. Multiple investigations have shown that SARS-CoV-2, especially in MIS-C, disrupts Treg stability and functionality [121,122]. In MIS C, activated CD4^+^ T cells are prevalent, whereas Tregs are destabilized [122]. A seminal study demonstrated that an aberrant Notch1/CD22 signaling pathway is activated in MIS C Tregs: Notch1 overexpression, driven by genetic and inflammatory stimuli, results in increased CD22 expression, which in turn induces mTORC1-mediated loss of Treg suppressive function [122]. MIS C seems to obstruct regulatory checkpoints on T cells, resulting in systemic inflammation [122]. This mechanistic insight reveals that the virus can directly undermine the regulating functions of the immune system [122]. Given that Tregs often uphold peripheral tolerance to allergens, their dysfunction post-COVID may reduce tolerance to normally innocuous antigens [120,121].

In the absence of MIS-C, SARS-CoV-2 may transiently alter Treg levels [123,124]. In adults, COVID-19 often manifests with lymphopenia and reduced Treg levels during the acute phase of the disease [123]. If Tregs are insufficient during recovery, the threshold for allergen-specific Th2 responses may diminish, facilitating a more vigorous response to allergens [121]. Moreover, the re-expanding Treg population may exhibit a changed epigenetic landscape, potentially affecting its functional capacity [125]. In children with increased naïve Treg fractions, initial strength may protect against immediate allergies; but, ongoing signals (e.g., residual IL-6/IL-10) could skew future Treg differentiation towards a less suppressive phenotype [121,126].

## 8. Imbalances of Th1, Th2, and Th17 Cells and Atopic Predisposition

A fundamental principle in allergic predisposition is the balance of T helper subsets [127,128]. Classical allergy is defined by Th2-dominated immunity (IL-4, IL-5, IL-13, eosinophils, IgE), whereas antiviral immunity is Th1-biased (IFN-γ, IL-2). Viral infections might transiently augment Th1 responses, potentially suppressing Th2 responses; however, extended or abnormal responses may later undermine tolerance and foster Th2 dominance [129]. Recent immunoprofiling of pediatric COVID-19 indicates a complex situation [130,131]. Children with MIS-C exhibit a pronounced Th1/Th17 cytokine profile [130,132]. Serum IFN-γ and its chemokines CXCL9 and CXCL10 are markedly enhanced in MIS-C compared to moderate pediatric COVID or healthy controls, in conjunction with increased levels of IL-6, IL-10, and IL-17 [130,132]. This indicates a hyperinflammatory state mediated by Th1 and Th17 pathways [130,132]. Notably, Th2-associated chemokines (e.g., CCL22) are reduced in MIS-C, suggesting a lack of standard counter-regulation [133]. In conclusion, MIS C redirects the immune response towards Th1/Th17 predominance [130,132].

Pediatric research demonstrates that severe SARS-CoV-2 infection can elevate levels of both pro-inflammatory and Th2 cytokines [134]. A review indicated that MIS-C and severe pediatric COVID are associated with increased levels of IL-1β, IL-6, IL-10, and the Th2 cytokine IL-13 [134]. Thus, whereas Th1 signals may rise, there may also be simultaneous increases in particular Th2-type cytokines [134]. This disordered pattern may stimulate atopic circuits [135]. Interleukin 13 is a crucial modulator of allergic inflammation, airway hyperreactivity, and immunoglobulin E class switching. The increase in post-infectious inflammation may consequently elevate the risk of acquiring allergies subsequently [129,135]. An existing atopic condition may partially influence the response to SARS-CoV-2 [136,137]. A study of asthmatic children revealed that individuals with elevated allergen-specific IgE or Th2 asthma exhibited diminished SARS-CoV-2 antibody and T cell responses [136]. This suggests that a Th2 dominating environment (allergic asthma) may diminish antiviral Th1 immunity [136]. Infection with SARS-CoV-2 may disrupt the immunological set point, causing infants with an atopic tendency to more easily revert to Th2/asthmatic phenotypes [137]. The observations indicate that SARS-CoV-2 can elicit a Th1/Th17 skewed, pro-inflammatory response that surpasses normal control, whereas sustained Th2-promoting signals such as IL-13 may prepare the post-viral environment for atopy [134,138,139]. The simultaneous occurrence of IL-10 and IL-13 in MIS-C is significant, as IL-10 reduces inflammation while also facilitating IgE production [138].

Temporary disturbance of the Th1/Th2/Th17 balance, along with an inability to re-establish homeostasis, may decrease the threshold for allergy sensitization [134,135]. Key immunological alterations following pediatric SARS-CoV-2 infection include: (1) upregulation of Th1/IL-17, with MIS-C samples exhibiting elevated levels of IFN-γ, CXCL9/CXCL10, IL-6, IL-17, and TNF-α compared to uncomplicated cases; (2) modified Th2 signaling, characterized by increased IL-13 in severe disease, decreased CCL22 in MIS-C, and the potential elicitation of virus-specific IgE; and (3) impaired immune checkpoints, where Th1 and Th17 activation coincides with a loss of regulatory control, creating a milieu conducive to Th2 auto- or allo-reactivity [130,132,134,139].

## 9. Cytokine Pathways and Immune Networks

Airway epithelial alarmins (IL-33, TSLP) and Th17 cytokines (IL-17A/F) affect the response to SARS-CoV-2 in allergic pediatric patients [140]. IL-33 is released by damaged epithelium and significantly enhances type 2 immunity (eosinophilia, IL-5/IL-13 production) via the activation of ILC2s and Th2 cells [141]. In viral infections, IL-33 recruits NK and CD8^+^ T cells, although its effects are context-dependent [142]. Increased serum IL-33 has been linked to worse COVID-19 pneumonia [143]. In allergic (T2 high) airways, IL-33 orchestrates Th2-mediated repair responses, promoting TGF-β and collagen deposition generated by IL-4/IL-13, whereas in non-allergic lungs, IL-33 may exacerbate an IFN-γ-driven cytokine storm [144]. IL-33 also reduces interferon production by plasmacytoid dendritic cells through TLR7 desensitization, potentially undermining initial antiviral defenses [145].

TSLP is an auxiliary epithelial alarmin that is activated by virus-induced NF-κB signaling. Infections caused by respiratory syncytial virus and rhinovirus in neonates significantly increase TSLP, leading to the polarization of dendritic cells and ILC2s towards type 2 inflammation [146,147]. Inhibition of TSLP by tezepelumab markedly diminishes virus-induced IL-33, Th2 cytokines (IL-4, IL-13), and IL-17A, while maintaining interferon responses [148]. Consequently, TSLP is positioned upstream of the IL-33/IL-25 cascade, orchestrating a combined Th2/Th17 airway response to viral stimuli [149]. IL 17A and IL 17F, derived from Th17 cells, are intimately associated with neutrophilic inflammation [150]. Severe pediatric viral infections, such as RSV, exhibit significant elevations in lung IL-6, IL-23, and IL-17. The experimental neutralization of IL-17 in RSV infection reduces mucus hypersecretion and enhances CD8^+^ T cell IFN-γ responses [151]. During COVID-19, IL-17F is significantly enhanced in critical illness and enhances endothelial ICAM-1 expression and neutrophil adhesion through ERK/p38 signaling [152]. In airways affected by asthma, IL-17 is significantly associated with heightened expression of SARS-CoV-2 entrance proteases; bronchial IL-17 upregulates epithelial ACE2/TMPRSS2. In contrast, the type 2 cytokines IL-4 and IL-13 typically diminish ACE2 expression [153,154]. In summary, IL-17 exacerbates pro-inflammatory lung injury and may facilitate viral entry, while IL-33 and TSLP increase Th2-mediated remodeling and attenuate innate interferon responses [140,145,148,154].

**Figure 4:**
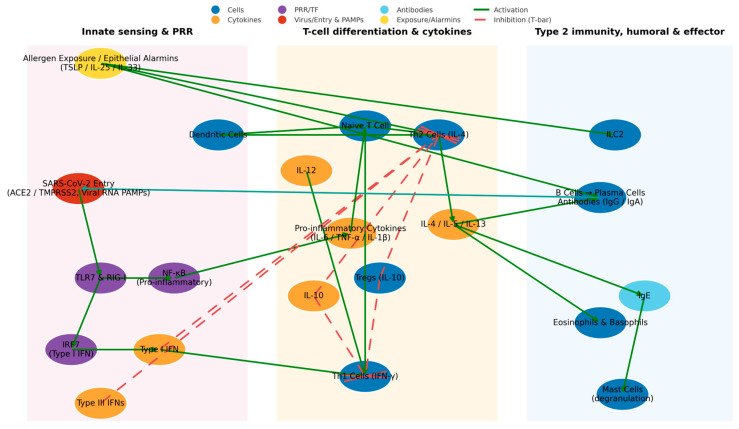
Network model of innate sensing, T-cell polarization, and effector responses at the interface of viral infection and allergen exposure. Nodes are grouped into three modules (light shading): Innate sensing and PRR (left), T-cell differentiation and cytokines (center), and Type-2 immunity, humoral and effector (right). Node colors denote category: cells (blue), cytokines (orange), PRR/transcription factors (purple), virus/entry and PAMPs (red), antibodies (sky blue), and exposure/alarmins (yellow). Edges encode regulation: solid green arrows = activation; dashed red arrows with T-bar heads = inhibition. Allergen exposure induces epithelial alarmins (TSLP/IL-25/IL-33) that activate ILC2 and promote Th2 cells and IL-4/IL-5/IL-13, culminating in B-cell class switching (IgE) and eosinophil/mast-cell effector functions; IL-4/IL-5/IL-13 also enhance plasma-cell responses. Viral RNA sensed via TLR7/RIG-I activates NF-κB (→ IL-6/TNF-α/IL-1β) and IRF7 (→ type I IFN), with type I IFN and IL-12 favoring Th1 polarization. Type III IFN and IL-10 dampen Th2 and inflammatory outputs; Tregs inhibit both Th1 and Th2. IgG/IgA neutralizing antibodies reduce SARS-CoV-2 entry (ACE2/TMPRSS2). Arrow direction reflects net influence and does not imply direct physical interaction. Abbreviations: PRR, pattern-recognition receptor; PAMPs, pathogen-associated molecular patterns; IFN, interferon; NF-κB, nuclear factor κB; IRF7, interferon regulatory factor 7; ILC2, group-2 innate lymphoid cell; TSLP, thymic stromal lymphopoietin; ACE2, angiotensin-converting enzyme 2; TMPRSS2, transmembrane protease serine 2; Ig, immunoglobulin; TNF-α, tumor necrosis factor-α.

Viral infections also elicit unique chemokine profiles [155]. SARS-CoV-2 infection triggers a cytokine storm characterized by increased levels of CXCL10/IP-10, CCL2/MCP-1, and IL-8, which recruit monocytes, neutrophils, and Th1 cells [145]. Detailed immunological pathways activated during SARS-CoV-2 infection and associated with allergy are presented in Figure 4.

In contrast, chronically allergic airways demonstrate elevated baseline levels of Th2 attractant chemokines, including CCL17/TARC, CCL22/MDC, and exotoxins [156]. Epithelial IL-33 further promotes eosinophil survival and the secretion of CXCL8 and CCL2, while interferon-stimulated genes induce CXCL10 and associated chemokines during SARS-CoV-2 infection [157]. Children with allergies generally exhibit diminished ACE2/interferon pathways and increased type 2 tone; however, changes in chemokines have not consistently provided protection or indicated risk [157]. 

Consequently, virus entry and inflammation in allergic lungs are regulated by the equilibrium between type 1 and type 2 alarmin signals [158,159] (Table 1).

## 10. Implications for Allergy Development and Persistence

Cohort studies, predominantly concerning adults, demonstrate an increased incidence of new-onset asthma and allergic rhinitis subsequent to COVID-19 [160,161,162]. Similar tendencies may emerge in youngsters, especially if their early immunological education was impaired [163]. The interaction of increased Th2 cytokines (IL-13), reduced Treg functioning, and an imbalanced microbiota may promote the development of IgE responses to environmental allergens [135,164,165]. Tissue remodeling due to lung or gastrointestinal inflammation may expose new self-antigens, potentially triggering food or drug allergies [144,164].

Children with pre-existing allergies may have diminished control or increased chronicity following COVID-19 [136]. When the airway epithelium is impaired by infection, the tolerance for asthma triggers decreases. The lasting recollection of virus-specific inflammation may “cross-prime” allergen-specific memory cells [155]. Moreover, trained innate immunity can exacerbate allergic inflammation following re-exposure [165]. Once sensitization occurs following COVID, it may exhibit an atypical resistance to tolerance induction [165]. SARS-CoV-2 induces long-lasting T and B cell memory in several convalescents [139]. The impact on long-term allergen memory, however, remains ambiguous [129].

In an inflammatory environment characterized by Th1/Th17 signals, memory T cells may be less predisposed to assume Th2 phenotypes [128]. However, allergen exposure during times of diminished tolerance can still establish strong Th2 memory [127]. Dysregulated germinal center activity in severe COVID-19 promotes polyreactive and autoreactive B cells, perhaps encompassing IgE-secreting clones [166].

SARS-CoV-2 significantly alters cytokine profiles associated with atopic sensitization [155]. Traditionally, Th1 cytokines (IL-12, IFN-γ) inhibit Th2 differentiation, while Th2 mediators (IL-4, IL-5, IL-13, IL-9) promote IgE synthesis and eosinophilia [128,167,168]. Research on COVID-19 indicates a significant type 2 bias [164]. Post-mortem lung tissues from fatal cases exhibit elevated levels of IL-4/IL-13 and a proliferation of M2 macrophages, indicating a Th2-dominated pathophysiology [164]. Children exhibit less pro-inflammatory responses that are comparatively Th2-oriented relative to adults, resulting in less severe illness. IL-17A and IFN-γ have an inverse correlation with age in mild pediatric infections, suggesting maintained IL 17/IFN activity in conjunction with a Th2 bias [132].

Th2 cytokines are closely associated with allergies. IL-4 and IL-13 facilitate IgE class flipping, while IL-5 promotes eosinophil survival [127]. IL-13 exacerbates SARS-CoV-2 pulmonary damage, whereas its neutralization alleviates the disease in vivo [157]. Airway “alarmins” IL-33, IL-25, and TSLP, secreted by virus-damaged epithelium, enhance ILC2 and Th2 pathways [140,146]. SARS-CoV-2 stimulates the production of IL-33, which facilitates the release of IL-5 and IL-13 [140,163]. Continued IL-33 generation by PBMCs following infection is associated with CD4^+^ T cell activation [163]. TSLP coordinates extensive type 2 signaling among dendritic, T, B, and innate cells [146,147]. Consequently, acute infection can establish a niche abundant in IL-4, IL-13, and IL-5, driven by alarmins, which promotes a Th2 bias [164,167]. In contrast, type I/II interferons activate antiviral defense mechanisms.

Children demonstrate rapid IFN-α/β production due to elevated basal levels of MDA5/RIG I and ISG expression [131]. SARS-CoV-2 undermines these pathways, postponing interferon release by approximately 48 h [155]. Older persons frequently exhibit diminished interferon activity and the presence of anti-interferon autoantibodies, which predisposes them to severe illness. Severe instances exhibit elevations in TNF-α and IL-1β [155], while paradoxically elevated IL-10 may indicate Treg dysfunction [134]. GM CSF increases, stimulating granulocytes and macrophages, hence exacerbating injury [154]. SARS-CoV-2 enhances type 2 and inflammatory mediators (IL-4, IL-13, IL-5, IL-6, TNF-α) while temporarily activating interferons (IFNs) [155,164]. Disrupted cross-regulation—wherein IFN-γ typically inhibits Th2 and IL-4/IL-13 constrain Th1—can pivot immunity towards Th2 predominance, decreasing the threshold for allergy sensitization [128,159].

## 11. Key Immune Cell Players

Conventional myeloid dendritic cells (DCs) acquire SARS-CoV-2 antigens and activate naïve CD4^+^ and CD8^+^ T cells, while plasmacytoid DCs (pDCs) primarily generate type I interferons through TLR7/9 recognition. The efficacy of dendritic cell (DC) function is crucial for immunological tolerance; however, SARS-CoV-2 has the capacity to infect or functionally deplete DCs [169]. Severe pediatric MIS-C exhibits a DC/monocyte activation profile indicative of dysregulated antigen presentation [139,169]. The absence of tolerogenic dendritic cell subsets, which typically promote the expansion of regulatory T cells, may therefore trigger unchecked inflammation [169]. A deficiency in pDC-mediated sensing, shown by harmful TLR7 mutations, predisposes individuals to life-threatening COVID-19 [170]. Alarmin cytokines in post viral-allergy are presented in Table 2.

Innate lymphoid cells type 2 (ILC2s) promptly release IL-5 and IL-13 in reaction to epithelial alarmins, including IL-33 and TSLP. The quantity of circulating ILC2s and activation markers (NKG2D, CD25) increased with the severity of COVID-19, alongside slight elevations in serum IL-5 and IL-13 levels [171]. Although amphiregulin provides immediate assistance in tissue healing, sustained IL-5/IL-13 production from ILC2 may predispose individuals to future atopic airway inflammation [171,172,173].

Mast cells and basophils, equipped with high-affinity IgE receptors, are crucial effector cells in allergic responses.

The spike protein of SARS-CoV-2 interacts with ACE2 on mast cells, inducing fast degranulation and the release of IL-1β and TNF, which exacerbate lung injury [173,174,175,176,177]. Human basophils exposed to SARS-CoV-2 or infected epithelium upregulate PD-L1 and release IL-13 following IL-3 priming, despite the absence of productive infection [174,178,179,180]. Innate IL-4/IL-13 sources can augment B cell assistance and facilitate eosinophilia during the viral response [174].

Eosinophil counts generally decrease (eosinopenia) during acute COVID-19, and persistent eosinopenia is associated with severe disease and mortality [175]. Post-recovery rebound eosinophilia may result in a hyper-responsive granulocyte reservoir that exacerbates subsequent allergic inflammation mediated by IL-5 and IL-13 [171,175].

Strong antiviral humoral immunity relies on T follicular helper (Tfh) cells that direct germinal center B cell responses [176]. Tfh subsets that produce IL-4 and IL-13 can facilitate IgE class shift, suggesting that bystander allergens encountered during recovery may be collected in an elevated Th2 environment [176]. Severe COVID-19 is characterized by disturbed germinal centers and the development of poly- or autoreactive B cells, perhaps involving IgE-producing clones.

Children develop long-lasting central memory and stem cell-like T cell reservoirs following SARS-CoV-2 infection [177,178]. Virus-specific memory T cells can, upon recall, induce the epithelial or innate release of IL-33, establishing a “memory alarmin” loop that connects adaptive recall to prolonged type 2 signaling [179]. This interaction may sustain a pro-atopic tissue milieu even after the acute infection has subsided [179].

## 12. Cytokine Cross-Regulation and Immune Checkpoints

Cytokine cross-talk modulates the balance between Th1 and Th2 immune responses [128,180]. Interferon γ and interleukin 12, generated by plasmacytoid dendritic cells (pDCs) and conventional dendritic cells type 1 (cDC1s), stimulate classical Th1 antiviral response. Nonetheless, there exist pathways that oppose each other; IL-12 inhibits OX40L-mediated Th2 polarization, whereas IL-4 decreases Th1 differentiation [169,181,182,183,184]. In contrast, IL-4, IL-5, and IL-13 secreted by Th2 cells and ILC2s enhance allergic inflammation [127,171]. The infection caused by SARS-CoV-2 can disrupt this equilibrium [155,185]. Prompt and robust type I IFN/IFN γ signaling in children accelerates viral clearance and inhibits IL-4/IL-5. When interferon responses are postponed or diminished, as observed in adults with neutralizing anti-IFN α/β autoantibodies, there is an excess of IL-6 and IL-1β, which subsequently promote Th2-skewing cytokines through IL-4R and epithelial alarmins.

Acute infection increases regulatory IL-10, but levels decline in protracted COVID, diminishing regulation of allergic inflammation [134,186]. In TSLP-activated dendritic cells, OX40L co-stimulation promotes Th2 TNF-α while inhibiting IL-10 [182]. IL-12 can counteract this bias, demonstrating how the cytokine environment determines the outcome [182,184]. Chronically elevated IL-4, as shown in atopy, further suppresses IFN-γ production [183]. Consequently, any increase in IL-4/IL-5 or decrease in IFN-γ induced by SARS-CoV-2 mutually suppresses antiviral Th1 defenses and promotes allergic responses [164,167]. Table 3 and Table 4 describe immune cell functions and mediators in allergy and in SARS-CoV-2 infection in both children and adults in acute and post-acute phase of SARS-CoV-2 infection.

Prolonged antigen exposure and promoting functional fatigue. Depleted cells produce diminished levels of IFN-γ and IL-2, while simultaneous Treg dysfunction compromises regulatory capacity [124,188]. Viral checkpoint modification, including the upregulation of PD-L1 on antigen-presenting cells, further restricts T cell assistance for B cells [180]. While certain epigenetic memories bolster future antiviral defenses, others reinforce regulatory or Th2-biased programs that predispose individuals to atopy [165,189]. Post-COVID monocytes exhibit heightened chromatin accessibility at the IL-10 and IFNG loci, demonstrating persistent reprogramming of regulatory and Th1 pathways [189]. These alterations establish a mechanistic connection between viral inflammation and the erosion of immunological tolerance [165].

FoxP3^+^ regulatory T cells typically inhibit inflammation and sustain allergen tolerance [110]. Severe COVID-19 is characterized by diminished circulating Tregs and decreased expression of FoxP3, IL-10, and TGF-β [122,124]. The absence of this regulating tone distorts immunity, leading to unregulated inflammation [120]. In children, transitory disruption of Treg during MIS-C can intensify hyperinflammation and hinder eventual tolerance to benign antigens [122].

PD-1 and CTLA-4 expressed on effector and regulatory T cells function as essential inhibitors of activation [177]. During acute infection, SARS-CoV-2 specific T cells transiently upregulate PD-1 while maintaining functionality [188]. Chronic stimulation or severe illness, however, induces persistent PD-1/CTLA-4 expression and true exhaustion, diminishing immunity [188]. Paradoxically, the overexpression of such checkpoints may inhibit bystander Tregs, potentially leading to post-recovery autoimmunity or allergy exacerbations [180,188]. In severe COVID-19, monocytes and dendritic cells exhibit elevated PD-L1 levels, a characteristic associated with compromised Treg production and a bias towards Th17 responses [169,180]. The typical cross-regulation, wherein IFN-γ inhibits Th2 cells and IL-4 suppresses Th1 responses, is disrupted during SARS-CoV-2 infection [128,155].

Increased levels of IL-6 and IL-1β impede the formation of FoxP3^+^ Treg, whereas reduced IL-10 fails to mitigate surges in IL-6 and TNF. Excessive Th17 proliferation exacerbates inflammation, as IL-17 stimulates the production of IL-6, G-CSF, and TNF-α [150,152]. This dysregulated environment collectively inhibits essential regulatory pathways while increasing type 2 mediators, mast cell activation, and eosinophilia, hence reducing the threshold for allergic sensitization following COVID-19 [164,171,173,187].

## 13. Molecular Mimicry and Autoantibodies

A broader spectrum of cytokines and antibodies—targeting IL-17, GM-CSF, annexin A2, type II collagen, among others—has also been recorded following infection [190,191,192,193]. Viral epitopes that mimic self-proteins can induce cross-reactive autoimmunity [191,194]. Research indicates molecular mimicry between SARS-CoV-2 antigens and endothelium or immune epitopes, resulting in autoantibody-mediated damage [112,132]. SARS-CoV-2 infection often induces a diverse array of autoantibodies [190,192]. Neutralizing autoantibodies against type I interferons are present in approximately 10–20% of patients with severe COVID-19, impairing antiviral defense. Wang et al. identified autoantibodies that can neutralize IL-33 and other alarmins, potentially biasing immunity towards Th2 pathways or modifying mast cell and eosinophil function [190]. Virus-induced autoantibodies can sustain inflammation or alter immunological networks over the long term [186]. Molecular mimicry and autoantibody production during SARS-CoV-2 infection collectively undermine peripheral tolerance and may decrease the threshold for subsequent allergy sensitization [127,165].

## 14. Trained Immunity, Epigenetic and Transcriptomic Reprogramming: Distinct Epigenetic Profiles Connecting COVID-19 to Atopic Susceptibility

Innate immune cells can acquire a “memory-like” or trained immunity state following robust stimulation [195,196]. SARS-CoV-2 infection epigenetically reprograms monocytes, NK cells, and ILCs, resulting in lasting chromatin alterations that endure for 4 to 12 months post-recovery [195]. Bulk and single-cell ATAC-seq experiments reveal significant hypomethylation and enhanced accessibility at interferon-stimulated gene loci in post-COVID monocytes, a pattern that coincides with markers of systemic lupus erythematosus and multiple sclerosis [196]. Severe acute infection also hastens the epigenetic age of immune cells, signifying accelerated biological aging of the innate compartment [197]. The permanent chromatin and methylation alterations indicate that innate cells maintain heightened responsiveness or faulty calibration long after recovery [195,196]. Trained monocytes/macrophages, upon secondary challenges, secrete elevated levels of IL-6 or GM-CSF, hence sustaining type 2 inflammation [195]. Dendritic cells originating from these progenitors exhibit enhanced costimulatory profiles, suggesting that bystander antigens presented in this inflammatory setting may promote sensitization instead of tolerance [182].

In pediatric patients, infected airway epithelium creates spatial cytokine gradients: IFN-α/β versus IL-6/TSLP [181]. Plasmacytoid dendritic cell-derived interferon enhances antiviral Th1 immunity, but TSLP-licensed dendritic cells upregulate OX40L and direct naïve CD4^+^ T cells towards an inflammatory Th2 phenotype (IL-4/IL-5/IL-13) [182]. Engagement of Toll-like receptors by SARS-CoV-2 can disrupt this equilibrium, enhancing OX40L and suppressing IL-12, so promoting Th2 polarization even amidst a viral infection [182,184].

“Omics” research validates the prolonged molecular reprogramming of myeloid cells: monocytes in the convalescent phase have open chromatin at IL-10 and IFNG, however exhibit heightened TNF production and diminished IL-10 release upon restimulation, indicative of a classic trained immunity signature [189]. Concurrent investigations of single cells and hematopoietic stem and progenitor cells demonstrate that early IL-6 signaling biases these cells towards myelopoiesis for an extended duration, populating the bloodstream with inflammatory progeny [195,196]. These progenitors generate monocytes that are baseline primed to secrete TNF-α and IL-1β, enhancing stimulation and facilitating Th2 priming upon re-exposure to allergens [195]. COVID-19-related CpG methylation alterations also connect viral infection to atopy: the cg04543273 locus inhibits LMAN2, a gene whose upregulation is associated with elevated circulating Th2 cells and is heightened following SARS-CoV-2 infection [197,198]. These findings demonstrate how virus-induced epigenetic modifications on immunological or epithelial cells might influence genes that favor type-2 immunity.

The ongoing IL-6 driven reprogramming of hematopoietic stem and progenitor cells, monocyte chromatin remodeling, and disease-specific CpG methylation collectively elucidate the increasing prevalence of post-COVID allergy diseases. Trained innate cells that excessively generate IL-6, GM-CSF, and TNF-α, together with TSLP/OX40L biased antigen presentation, diminish the threshold for Th2 priming, IgE class switching, and eosinophilic inflammation during subsequent allergen exposures [195,198]. Epigenetic/transcriptomic signatures post–COVID-19 are presented in Table 5.

## 15. Conclusions

SARS-CoV-2 infection in children perturbs the delicate balance of antiviral and allergic immune networks. Viral-induced epithelial damage, alarmin cytokine release, Th2 polarization, and trained immunity in monocytes converge with epigenetic and microbiome alterations to generate a milieu that may predispose to allergy. While some mechanisms, such as reduced ACE2 expression in atopic airways, suggest partial protection from viral entry, the overall picture indicates trade-offs that can lower thresholds for allergic sensitization.

While reduced ACE2 expression might transiently protect allergic children from viral invasion, the same immunological environment amplifies type-2 responses and lowers tolerance thresholds. Recognizing these dual effects is essential for understanding how SARS-CoV-2 can simultaneously confer partial protection against infection severity yet increase long-term predisposition to allergy.

Evidence from large multinational cohorts supports an increased risk of asthma and allergic rhinitis, yet findings remain inconsistent across regions, underlining the need for harmonized global surveillance. From a clinical perspective, these insights underscore several implications: (1) preventive strategies, including COVID-19 vaccination, which appears to mitigate allergy risk; (2) the potential role of emerging biologics targeting IL-33 or TSLP in reducing post-viral atopic sequelae; and (3) the importance of microbiome restoration approaches to rebalance epithelial-immune interactions. Future studies must integrate single-cell and epigenetic profiling with longitudinal follow-up of pediatric cohorts to delineate causality. By merging epidemiology with mechanistic immunology, clinicians may better identify children at risk and develop targeted interventions to prevent long-term allergy burden in the post-COVID era.

## Figures and Tables

**Table 1:** Cytokine regulation of SARS-CoV-2 receptors including allergic/type-2 cytokines vs. antiviral interferons.

Mediator	Allergy (Type 2 Asthma)	SARS-CoV-2 Infection	Effect on ACE2/TMPRSS2	References
IL-4	↑ IL-4 (Th2 cytokine)	Lower in antiviral state	Reduces ACE2 expression; little effect on TMPRSS2.	[28,30,40]
IL-13	↑↑ IL-13 (Th2 cytokine)	Lower in antiviral state	Reduces ACE2, increases TMPRSS2.	[30,56,73,74]
IFN-γ	↓ IFN-γ	↑ in viral response	Increases ACE2; modest effect on TMPRSS2.	[58,59]
TSLP/IL-33/IL-25	Produced by epithelium (alarmins)	↑↑ on infection/repair	Induce ILC2→ ↑IL-5/IL-13; indirect ↓ACE2 via IL-13.	[9,10,11,140,146,147]
TGF-β	↑ in chronic allergy remodeling	Varied (fibrosis role)	Indirect immunoregulatory; possibly ↑ACE2 via repair processes (speculative).	[127,144]

Abbreviations: The up (↑) and down (↓) arrows in the table caption are shorthand indicators of relative increase or decrease in the levels of a cytokine/mediator under different conditions (e.g., allergy vs. viral infection). ↑ (single up arrow) → indicates a moderate increase (above baseline). ↑↑ (double up arrow) → indicates a strong or pronounced increase. ↓ (down arrow) → indicates a decrease (below baseline).

**Table 2:** Alarmin cytokines in post-viral allergy. Alarmins are epithelial-derived signals that kick-start type-2 immunity. For example, IL-33 and IL-25 released during SARS-CoV-2–induced cell damage directly activate ILC2s, while TSLP licenses DCs to drive Th2 differentiation. These alarmins thereby enhance IL-4/IL-5/IL-13 responses and eosinophilic inflammation following viral infection.

Alarmin Cytokine	Induction (Triggers)	Downstream Immune Effects	References
IL-33	Released by necrotic epithelial/endothelial cells and damaged airway epithelium; upregulated in COVID-19.	Binds ST2 on ILC2 and Th2 cells, inducing IL-5 and IL-13 production and eosinophil recruitment; also activates mast cells. In SARS-CoV-2 infection, elevated IL-33 correlates with lung hyperinflammation.	[140,143,144,157]
IL-25 (IL-17E)	Produced by airway tuft and epithelial cells upon viral infection or allergen exposure.	Activates ILC2 to secrete IL-5, IL-13 (and some IL-4), promoting eosinophilia and mucus production. Synergizes with IL-33 and TSLP in driving type-2 immunity.	[9,12,147]
TSLP	Produced by airway and gut epithelia in response to viruses, allergens, or mechanical stress.	Primes myeloid DCs to express OX40L (and upregulate IL-4Rα), polarizing naive CD4^^+^ T cells into Th2 cells. Also acts directly on basophils, mast cells and ILC2 to augment type-2 cytokine release.	[146,147,148,149]
HMGB1	Nuclear alarmin released by necrotic cells or secreted by activated macrophages.	Enhances dendritic cell maturation and Th2 priming (through RAGE/TLR4) and can promote airway hypersensitivity.	[150]
TSLP/IL-25/IL-33 (triad)	Often act in concert when epithelial damage occurs.	Jointly potently activate ILC2 and prime Th2 immunity even without antigen presentation. (ILC2 activated by any of these alarmins produce IL-5/IL-13, as in viral asthma models.)	[9,10,146]

**Table 3:** Immune cell functions and mediators in allergy and in SARS-CoV-2 infection. This synthesis contrasts key leukocyte roles. For example, TSLP-activated DCs express OX40L to drive Th2 differentiation, whereas pDCs produce IFN-α for viral defense. Mast cells and eosinophils are central effectors in allergy, but they also become aberrantly activated by SARS-CoV-2 (mast cells release histamine and cytokines in COVID-19). ILC2s are innate sources of IL-5/IL-13 (triggered by IL-33, IL-25, TSLP) that amplify Th2 responses.

Cell Type	Allergy Role	SARS-CoV-2 Role	References
CD4^+^ Th2	Produce IL-4, IL-5, IL-13; help B cells class-switch to IgE; drive eosinophil/mast cell recruitment.	(Typically low in acute infection) Can arise if allergic bias; not major antiviral role.	[127,128,129]
CD4^+^ Th1	(Baseline) Minor in allergy; secrete IFN-γ, IL-2 for macrophage activation.	Essential for viral clearance: produce IFN-γ, TNF-α; support CD8^+^ CTLs.	[128,155,181]
CD8^+^ T cells	(Baseline) Limited direct role in allergy.	Kill infected cells; produce IFN-γ/TNF; express granzyme B.	[37,176]
Tregs (CD25^+^FoxP3^+^)	Suppress allergic inflammation via IL-10, TGF-β; maintain tolerance to allergens.	Expand during COVID-19 (especially in children); can dampen immunopathology and aid resolution.	[120,122,124]
Th17 Cells	Produce IL-17; contribute to chronic airway inflammation and neutrophilia in severe asthma.	Contribute to hyperinflammation in MIS-C/COVID-19; secrete IL-17 (elevated in MIS-C).	[130,150,151]
Tfh Cells	Support IgE class switching by B cells in allergy; produce IL-4.	Support germinal center formation for anti–SARS-CoV-2 antibodies.	[176,177]
B cells/Plasma cells	Class-switch to IgE/IgG4 in allergy (IL-4 dependent); produce allergen-specific IgE.	Produce IgM/IgG/IgA against viral antigens; neutralizing antibodies.	[162,166,167]
Conventional DCs	Subsets: cDC2 present allergens to Th2 cells (via OX40L if TSLP-activated).	cDC1 cross-present viral antigens to CD8^+^ T cells; produce IL-12 to drive Th1.	[169,182]
Plasmacytoid DCs (pDCs)	Not involved in allergy.	Produce large amounts of type I/III IFN upon sensing SARS-CoV-2; crucial for antiviral defense.	[170,181]
Mast Cells	Express FcεRI for IgE; degranulate to release histamine, proteases, TNF-α, IL-4/IL-13; orchestrate allergic hypersensitivity.	Express TLRs; activated by viral RNA or complement; release histamine and pro-inflammatory cytokines in COVID-19.	[43,173]
Basophils	Circulate with FcεRI-bound IgE; release IL-4 and IL-13 upon allergen encounter; support Th2 polarization.	Can be activated during viral infection; possibly produce IL-4.	[174,180]
Eosinophils	Recruited by IL-5; release major basic protein and eosinophil peroxidase, contributing to tissue damage in allergy.	Decreased in acute COVID-19 (eosinopenia); but COVID patients can have eosinophil activation markers.	[32,61,175]
Neutrophils	Attracted by IL-8; contribute to late-phase allergic inflammation (release ROS, proteases).	First responders in COVID-19; release NETs and ROS, contribute to lung damage in ARDS.	[150,155]
NK cells	(Minor) Produce IFN-γ; may help contain low-level viral infections in allergic airways.	Kill virus-infected cells early; produce IFN-γ/TNF-α; their activity is often blunted by SARS-CoV-2.	[37,181]
ILC2 (Innate Lymphoid 2)	Produce IL-5, IL-13, IL-9 (after IL-25/IL-33/TSLP stimulation); potentiate Th2 immunity and tissue eosinophilia.	Activated by virus-induced epithelial alarmins; contribute to airway hyperreactivity even without allergen.	[60,171,172]
Macrophages	M2-type macrophages (IL-4/IL-13-driven) produce IL-10, TGF-β; support tissue remodeling in chronic allergy.	M1-type macrophages produce IL-1β, IL-6, TNF-α in viral infection; essential for pathogen clearance (but can be pathological if overactive).	[164,187]

**Table 4:** Comparative cytokine milieu in SARS-CoV-2 infection in pediatric vs. adult acute and post-acute phases. Pediatric acute COVID-19 (usually mild) elicits low systemic cytokines, whereas adults often show high IL-6/IL-1β during acute disease. In post-acute (PASC/long COVID), adults can show a profile of high IL-17 and IL-2 with low IL-4/IL-10. By contrast, most children returning to health have normal cytokine levels. (Arrows indicate relative changes: ↑↑: strong increase, ↑ moderate increase, ↔ no significant change, ↓ decrease. –: No data available.)

Cytokine	Pediatric (Acute)	Pediatric (Post-Acute)	Adult (Acute)	Adult (Post-Acute)	References
IL-6	↔ ~normal (low inflammation)	↔ (typically normal or slight ↑)	↑↑ (elevated, especially if severe)	↔ (return toward baseline unless PASC)	[108,111,155]
IL-1β	↔ (mild)	↔	↑ (elevated in severe cases)	↔ (usually normal post-recovery)	[111,155]
TNF-α	↔ (mild)	↔	↑ (elevated)	↔/↑ (can remain modestly ↑ in PASC)	[155,157]
IFN-γ	↑ (robust innate response)	↔	↑ (T cell response)	↔ (normalizing)	[37,181]
IL-2	↔ (baseline)	↔	↔ (baseline)	↑ (often elevated in long COVID)	[130,188]
IL-10	↔ (low in mild, ↑ if MIS-C)	↔ (normalizing)	↑ (feedback in acute)	↓ (often low in long COVID)	[111,134]
IL-4	↔ (no major change)	↔	↔ (not prominently induced)	↓ (low in long COVID)	[155,164]
IL-5, IL-13	↔ (baseline)	↔	↔ (baseline)	↔ (baseline or low)	[30,168]
IL-17	↔ (slight ↑ in MIS-C)	↔	↑ (elevated in severe)	↑ (markedly ↑ in PASC)	[130,151,152]
CCL11 (eotaxin)	–	–	↑ (inflammation)	–	[156]

**Table 5:** Epigenetic/transcriptomic signatures post–COVID-19. Studies of blood cells after SARS-CoV-2 infection reveal lasting molecular scars. For example, DNA methylation profiling shows altered methylation in IFN and antigen-presentation genes in monocytes. Single-cell ATAC-seq finds persistent chromatin openness at IL-10 and IFNG in convalescent monocytes. These changes constitute an “innate memory” imprint that can shift cytokine outputs. Certain COVID-linked CpG sites (e.g., in LMAN2) are also connected to Th2-skewed outcomes. Together, these markers indicate a reprogrammed immune landscape that may underlie loss of tolerance.

Epigenetic Marker/Change	Affected Genes/Pathways	Potential Impact on Immune Dysregulation	References
DNA methylation (monocytes)	Hypomethylation of interferon-related genes; changes at antigen-presenting genes.	Alters innate antiviral response (dampened IFN signaling) and antigen presentation, skewing subsequent adaptive responses.	[195,196]
Chromatin accessibility (monocytes)	Increased open chromatin at IL10 and IFNG loci.	Durable “memory” in monocytes leading to altered cytokine production (e.g., heightened IFN-γ/IL-10 potential) long after infection. Could bias balance of Th1/regulatory signals in tissues.	[189,195]
CpG methylation at LMAN2 (cg04543273)	cg04543273 (LMAN2 intron) hypomethylation suppresses LMAN2 expression; LMAN2 is upregulated in COVID-19.	Linked to increased Th2-cell abundance; suggests SARS-CoV-2–induced methylation may promote Th2 polarization and atopic dermatitis.	[94,198]
Histone modifications (general)	Reported global increase in H3K4me1, H3K27ac at inflammatory loci in myeloid cells (unpublished/analogous to sepsis models).	Facilitates rapid re-expression of pro-inflammatory genes upon stimulus, potentially exacerbating allergic inflammation.	[195,196]
HSPC transcriptional reprogramming	IL-6–driven upregulation of myelopoiesis genes in HSPCs.	Skews hematopoiesis toward inflammatory myeloid cells for months, embedding a pro-allergic innate bias in new monocytes/macrophages.	[195,197]

## Data Availability

No new data were created or analyzed in this study. Data sharing is not applicable to this article.
